# The Electrophysiological Features in X-Linked Charcot-Marie-Tooth Disease With Transient Central Nervous System Deficits

**DOI:** 10.3389/fneur.2018.00461

**Published:** 2018-06-27

**Authors:** Qingxian Wen, Longqiao Cao, Cun Yang, Yanchen Xie

**Affiliations:** ^1^Department of Neurology, Jining No. 1 People's Hospital, Jining, China; ^2^Department of Reproductive Medicine, Jining No. 1 People's Hospital, Jining, China; ^3^Department of Pediatrics, Jinning No. 1 People's Hospital, Jining, China; ^4^Department of Neurology, Washington Institute of Clinical Research, Vienna, VA, United States

**Keywords:** X-linked Charcot-Marie-Tooth disease, electrophysiological, white matter, transient, *GJB1*

## Abstract

**Background:** Electrophysiological examination plays an important role in the diagnosis of X-linked Charcot-Marie-Tooth disease (CMTX1) with transient central nervous system deficits. However, the electrophysiological features are seldom reported.

**Methods:** We reviewed and analyzed published reports to determine the electrophysiological features of CMTX1 patients with transient central nervous system deficits.

**Results:** A total of 21 CMTX1 patients with transient central nervous system deficits were found in 17 published case reports/series. The age of onset ranged from 0.5 to 18 years (mean 12.02 ± 0.78 years). All patients were male. Recurrent episodes of central nervous system deficits were reported in all 21 cases and resolved in periods ranging from several minutes to 3 days. All 20 patients who had MRIs at presentation had bilaterally symmetrical abnormal T2/Diffusion signals in the white matter without enhancement of gadolinium. All subsequent MRIs showed improvement or were within normal limits. The median motor nerve conduction velocity (MNCV), motor latencies, and compound muscle action potential (CMAP) amplitude were the most commonly measurable electrophysiological parameters (85.7%). All cases that had MNCV at presentation had slower and significantly decreased MNCV compared with the normal value (34.1 ± 1.10 m/s vs. 46.8±2.05 m/s, *P* < 0.0001; 95% CI, −17.4 to −7.92). The average variations of MNCV in median nerve, ulnar nerve, peroneal nerve, and tibial nerve were 22.0 ± 5.96%, 27.6 ± 11.9%, 25.9 ± 4.36%, and 27.3 ± 4.30%, respectively. All cases with measured sensory nerve conduction velocity (SNCV) at presentation had slower and significantly decreased SNCV compared with the normal value (35.3 ± 1.33 m/s vs. 47.7 ± 2.40 m/s, *P* < 0.001; 95% CI −18.2 to −6.46). The average variations of SNCV in median nerve, ulnar nerve, and sural nerve were 19.9 ± 8.24%, 25.2 ± 7.75%, and 23.2 ± 3.95%, respectively.

**Conclusion:** This case series serves as a reminder that electrophysiological examination should be included in the diagnosis of recurrent and episodic neurological deficit with white matter lesions. Median MNCV is a sensitive and valuable parameter to support the diagnosis of CMTX1 with transient central nervous system deficits.

## Introduction

Charcot-Marie-Tooth disease (CMT), also known as hereditary motor and sensory neuropathy or peroneal muscular atrophy, is a genetically and clinically heterogeneous group of inherited neuropathies (1). Clinically, it is characterized by progressive muscle weakness and atrophy, and distal sensory loss (2). X-linked CMT (CMTX1) is the second most common form of CMT, and it is caused by *gap junction protein beta-1* (*GJB1*, also known as *Cx32*) gene mutations. The phenotype of CMTX1 is typically characterized by a mixture of demyelinating and axonal features (3).

Transient central nervous system deficits associated with white matter abnormalities on magnetic resonance imaging (MRI) have been reported in patients with CMTX1 (4, 5). Electrophysiological examination plays an important role in diagnosis of CMTX1, particularly when there is a need to differentiate it from the diagnosis of stroke-like acute onset neurological deficits with white matter lesions (6) or episodic acute demyelinating encephalomyelitis (ADEM)-like illness (7). However, electrophysiological assessment in CMTX1 with transient central nervous system deficits is rare, and thus the electophysiological features are seldom reported.

Here we report a large series of CMTX1 patients with transient central nervous system deficits who had electrophysiological examination. We reviewed and analyzed the published reports with a primary goal of determining the electrophysiological features of CMTX1 patients with transient central nervous system deficits and discussing aids in diagnosis.

## Methods

### Search strategy and study screening

A systematic literature search was conducted on March 21, 2018 on PubMed (from January 1966) and Embase databases (from 1947). Key search terms used were: Charcot-Marie-Tooth, X-linked, gap junction or GJB1 or connexin, white matter or encephalomyel^*^ or central nervous or leukoencephal^*^, electrophysiol^*^ OR electromyo^*^ OR EMG OR motor nerve conduction velocity (MNCV) OR distal motor latencies OR compound muscle action potential (CMAP). A manual search of relevant review papers was also performed.

The current investigation was performed and reported according to the Preferred Reporting Items for Systematic Reviews and Meta-Analyses (PRISMA) guidelines (8). All citations and selected articles were read in full and rated on quality by two independent reviewers (Wen and Yang).

### Inclusion criteria, and exclusion criteria

The inclusion criteria were as follows: (1) X-linked Charcot-Marie-Tooth with transient central nervous system deficits; (2) electrophysiological examination was performed; (3) case report or case series. The exclusion criteria were as follows: (1) no adequate data reported or obtained by contacting the authors; (2) duplicate publications.

### Data extraction

**Figure 1:**
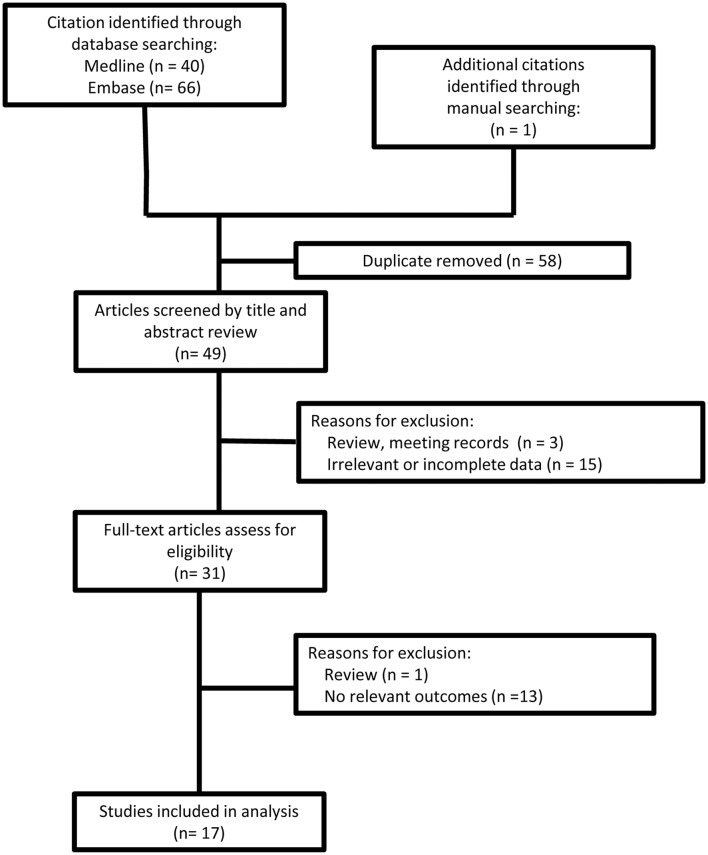
Flowchart of search strategy and study selection.

Search strategy and study selection are presented in Figure 1. A data extraction sheet was used to record study and patient demographic variables; family history; genetic mutation; and clinical, MRI and electrophysiological findings. Data were extracted and verified by two investigators (Wen and Cao).

### Statistical analysis

Statistical analysis was performed with GraphPad Prism 5 software (GraphPad Software Inc., La Jolla, CA) and SPSS Statistics 13 (SPSS Corporation, Chicago, IL). The continuous variables are presented as means with 95% confidence intervals (CI). A two-tailed comparison with *P* < 0.05 was considered statistically significant. Differences between groups were analyzed by the independent sample *t*-test or the Mann-Whitney *U*-test (for nonparametric comparisons), for the continuous variables.

## Results

### Search results and demographic characteristics

A total of 21 CMTX1 patients with transient central nervous system deficits were found in 17 case reports/series published between 2001 and 2016 (4–7, 9–21). Demographic, clinical, MRI, genetic, and electrophysiological data of 21 cases from the literature were reviewed (see Tables 1–3, Supplement Table 1).

**Table 1:** Demographic characteristics, family history, and mutation in CMTX1 with transient central nervous system deficits.

**No. of patient**	**Year**	**Country**	**Age at onset**	**Age at examination**	**Family history**	**Diagnosis of families**	**Nucleotide transition**	**Amino acid substitution**	**References**
1	2001	Greece	10	21	Brother, mother	Brother, same disease; Mother, asymptomatic pes cavus	C164T	Thr55Ile	Panas et al. 5
2	2001	Greece	12	19	Brother, mother	Brother, same disease; Mother, asymptomatic pes cavus	C164T	Thr55Ile	Panas et al. 5
3	2003	Germany	10	12	Brother, mother	Brother, same disease; Mother, CMTX	304-306delGAG		Hanemann et al. 9
4	2003	Germany	0.5	19	Brother, mother	Brother, same disease; Mother, CMTX	304-306delGAG		Hanemann et al. 9
5	2010	Italy	14	14	Mother	CMT	G164A	Arg164Gln	Fusco et al. 10
6	2011	Japan	13	15	Mother	CMT	397delT		Sakaguchi et al. 11
7	2012	China	14	17	Maternal grandfather	CMT		Asn54Ser	Zhong et al. 12
8	2014	Korea	14	14	Mother	Asymptomatic pes cavus	G3T	Met1Ile	Kim et al. 7
9	2014	Italy	16	29	Mother	clinical CMT	297_298insCAA	Gln99_His100inGln	Sagnelli et al. 13
10	2014	China	15	15	Mother	Asymptomatic pes cavus	T278G		Zhao et al. 14
11	2014	USA	12	12	Mother	Asymptomatic pes cavus	T98A	Ile33Asn	McKinney et al. 15
12	2015	USA	13	20	Mother and maternal relatives	CMT	T467G	Leu156Ala	Wu et al. 6
13	2015	India	8	19	Brother, mother	CMT	G425A	Arg142Gln	Kulkarni et al. 16
14	2016	China	18	24	Maternal grandfather	CMTX	T445C	Pro149Leu	Xie et al. 17
15	2002	Netherlands	10	14	Mother	CMT	C>T	Arg164Trp	Schelhaas et al. 18
16	2008	USA	13	15	Brother, mother	CMTX		Val139Met	Halbrich et al. 19
17	2008	USA	15	16	Brother, mother	CMTX		Val139Met	Halbrich et al. 19
18	2010	USA	10	10	Maternal grandmother	CMT	codon 22	Arg22Glu	Rosser et al. 20
19	2011	Canada	11	11	Mother	CMT	196G196A	Asn66Asn	U-King-Im et al. 21
20	2013	USA	14	14	NA	NA	C260G	Pro87Leu	Al-Mateen et al. 4
21	2013	USA	10	17	Mother	CMT	G477A	Val139Met	Al-Mateen et al. 4

*CMTX, X-linked Charcot-Marie-Tooth disease*.

### Demographic characteristics, family history, and mutation

Demographic characteristics, family history, and mutation in CMTX1 with transient central nervous system deficits are shown in Table 1. The age of CNS disturbance onset ranged from 0.5 to 18 years old with a mean of 12.02 ± 0.78 years. The age range of examination was 10–29 years old, with a mean ± standard deviation (SD) of 16.52 ± 0.99 years. All patients were male. Seven patients were from Europe, six from Asia, and eight from North America. Family histories were found in all cases. Two brothers were reported as CMTX1 with transient central nervous system deficits with the same mutation in *GJB1* gene in 3 families. All the other families had proband's mother or maternal relatives with CMT or asymptomatic pes cavus.

### Clinical and MRI features

Clinical and MRI features in CMTX1 with transient central nervous system deficits are shown in Table 2. Recurrent episodes of the central nervous system deficits were reported in all 21 cases and resolved in periods ranging from several minutes to 3 days. All 21 patients (100%) had transient weakness of limbs, 17 patients (80.95%) had transient dysarthria (10/21), dysphagia (3/21), or both (4/21); 6 patients (28.57%) had numbness, and 2 patients (9.52%) had ataxia. The transient central nervous system deficits presented after a fever in 9 cases (42.86%), exercise in 4 patients (19.05%), travel to high altitude in 2 patients (9.52%), and vomiting and diarrhea in 1 patient (4.76%). All 20 patients who had MRI at presentation had bilateral abnormal T2/Diffusion signal in the white matter. The other patients without MRI presented with recurrent episodes of transient neurological dysfunction on two consecutive days. Investigations included normal results for brain computed tomography (CT) scan, CT angiogram, lumber puncture (*n* = 19, Tables 1, 2).

**Table 2:** clinical and MRI features in CMTX1 with transient central nervous system deficits.

**No. of patient**	**Limb weakness**	**Dysarthria/ dysphagia**	**Numbness**	**Ataxia**	**Predisposing factor(s)**	**Duration to recovery**	**Clinical characteristics**	**Bilaterally symmetric white matter lesions on MRI**	**Enhancement**	**MRI improvement**	**Time of MRI follow-up**
1	Weakness of limbs	Dysarthria, dysphagia				5 h to 3 days	Recurrent episode	Yes	NA	NA	NA
2	Weakness of limbs						Recurrent episode	Yes	NA	NA	NA
3	Weakness of limb	Dysarthria, dysphagia			Exercise, high altitude	1, 3 h	Recurrent episode	Yes	no	Yes	3 months
4	Weakness of limb	Dysarthria, dysphagia			Fever	1, 24 h	Recurrent episode	Yes	no	Yes	6 months
5	Weakness of limbs	Dysarthria	Numbness	Ataxia	Fever	12–24 h	Recurrent episode	Yes	NA	Yes	2 months
6	Paresis of left arm	Dysphagia	Numbness		Vomiting and diarrhea	3 h	Recurrent episode	Yes	NA	Yes	11 days
7	Right hemiparesis	Dysarthria			Fever	5–7 h	Recurrent episode	Yes		Yes	3 months
8	Weakness of limbs	Dysarthria				I h	Recurrent	Yes	No	NA	NA
9	Right hemiparesis				Exercise	2 h	Recurrent	Yes	NA	Yes	18 months
10	Weakness of limbs	Dysphagia			Exercise, fever	2 h	Recurrent	Yes	ND	Yes	2 months
11	Left arm weakness	Dysphonia, Dysphagia				3 days	Recurrent, episode	Yes	No	Yes	2 months
12	Left facial weakness	Dysarthria	Numbness			3 h	Recurrent, episode	Yes	No	Yes	3 months
13	Weakness of limbs	Dysarthria	Numbness			20, 30 min and 10 h	Recurrent episodes	Yes	NA	Yes	1 month
14	Weakness of limbs	Dysarthria				4 h	Recurrent episodes	Yes	no	Yes	2 months
15	Weakness of both limbs	Dysarthria, dysphagia				6 h	Recurrent episode	Yes		Yes	2 months
16	Weakness of limbs	Dysarthria			Fever	8 h	Recurrent episode	ND	ND	NA	NA
17	Weakness of limbs	Dysarthria			Fever	4 h	Recurrent episode	Yes	no	Yes	3 months
18	Right upper extremity weakness		Numbness	Ataxia		3 h	Recurrent episode	Yes	No	Yes	6 months
19	Weakness of right upper limb	Aphasia			Fever	2.5 h	Episode	Yes	Yes	Yes	3 months
20	Right hand weakness		Numbness		High altitude, fever	6h-3days	Recurrent	Yes	No	Yes	2 months
21	Weakness of limbs	Dysarthria			Fever	Several min, 90 min, 12 h	Recurrent episode	Yes	No	Yes	2 months

*CMTX, X-linked Charcot-Marie-Tooth disease*.

All 11 cases with administration of gadolinium during the brain MRI reported no enhancement. Subsequent MRI results in 17 cases were within normal limits or showed improvement. The follow-up MRIs were performed from 11 days to 18 months after the episode of the disease, median 2 months.

### Electrophysiological features

Of the 21 electrophysiological examinations, 14 cases had detectable electrophysiological parameters, and 7 cases had normal laboratory values (9–12, 14, 15). Supplementary Table 1 summarizes 21 electrophysiological examinations in CMTX1 patients with transient central nervous system deficits. The changes in electrophysiological parameters were compared with the normal values, and the variation with respect to normal values is shown in Table 3.

**Table 3:** Variations of electrophysiological examination in CMTX1 with transient central nervous system deficits.

**No. of patient**	**Median nerve**					**Ulnar nerve**					**Peroneal nerve**			**Tibial nerve**			**Sural nerve**	
	**CMAP amplitude (%)**	**Distal latency (%)**	**MNCV (%)**	**SNAP amplitude (%)**	**SNCV (%)**	**CMAP amplitude (%)**	**Distal latency (%)**	**MNCV (%)**	**SNAP amplitude (%)**	**SNCV (%)**	**CMAP amplitude (%)**	**Distal latency (%)**	**MNCV (%)**	**CMAP amplitude (%)**	**Distal latency (%)**	**MNCV (%)**	**SNAP amplitude (%)**	**SNCV (%)**
3														Normal		−27	−66.7	
4														Normal		−28.6	Normal	
5	−60	+100	−22.2	−33.3	−20				−50	−24.4	−20	+25	−17.5	−6	+50	−17.5		
6	−80.3	+26.1	−25.5	−71.4	−15.1	−58	Normal	−25.9	−55.1	−12.2	−98	+1.5	−27.9	−87.2	+19.3	−14.9	−54.1	−19.2
7	−86.7	+231	−10	−26.1		−86.7	+171	−8	−58.7		−96.2	+540	−32.2	−85.6	+586	−44.2		
10	−92	+40	−38	−92	−42	Normal	Normal	−49	−89	−39				−98	Normal			
11	−71.4	+25	−23.3	Normal	−2.5									−34.5	+13.3	−31.5	−47	−27.1
Average	−78.1 ± 5.68	+84.5 ± 39.2	−22.0 ± 5.96		−19.9 ± 8.24			−27.6 ± 11.9	−63.2 ± 8.78	−25.2 ± 7.75	−71.4 ± 25.7	+189 ± 176	−25.9 ± 4.36			−27.3 ± 4.30		−23.2 ± 3.95

*CMTX, X-linked Charcot-Marie-Tooth disease; CMAP, compound muscle action potential; MNCV, median motor nerve conduction velocity; SNAP, sensory nerve action potential (SNAP); SNCV, sensory nerve conduction velocity*.

All nerve conduction examinations performed at presentation showed marked slowing of conduction velocity. The median MNCV, CMAP amplitude, and motor latencies were the most commonly measurable electrophysiological parameters (85.7%). Median MNCVs were all below the normal value, and mean ± SD MNCVs of the 12 cases were 37.7 ± 1.22 m/s (range 32–48.5 m/s).

All cases that had MNCV at presentation had slower and significantly decreased MNCV compared with the normal value (34.1 ± 1.10 m/s vs. 46.8 ± 2.05 m/s, *P* < 0.0001; 95% CI, −17.4 to −7.92). The average variations of MNCV in median nerve, ulnar nerve, peroneal nerve and tibial nerve were 22.0 ± 5.96%, 27.6 ± 11.9%, 25.9 ± 4.36%, 27.3 ± 4.30%, respectively (Table 3). All cases that had SNCV at presentation had slower and significantly decreased SNCV compared with the normal value (35.3 ± 1.33 m/s vs. 47.7 ± 2.40 m/s, *P* < 0.001; 95% CI −18.2 to −6.46). The average variations of SNCV in median nerve, ulnar nerve and sural nerve were 19.9 ± 8.24%, 25.2 ± 7.75%, and 23.2 ± 3.95%, respectively.

Distal latency was observed. All cases that had distal latency for median nerve and peroneal nerve had prolonged latency compared with the normal value, and mean changes were 84.5 ± 39.2% and 189 ± 176%, respectively. There was normal distal latency for the ulnar nerve in 2 cases (11, 14) and for tibial nerve in 1 case (14).

For the CMAP amplitude, all cases that had median nerve and peroneal nerve examination had reduced amplitude compared with normal values. Mean changes were 78.1 ± 5.68% and 71.4 ± 25.7%, respectively. However, the tibial nerve in 2 cases (9) and the ulnar nerve in 1 case had normal CMAP amplitude (14).

Most cases had reduced sensory nerve action potential (SNAP) amplitude, except 1 case in the median nerve (15) and 1 case in the sural nerve (9).

### Brainstem auditory evoked potentials, visual evoked potentials, and somatosensory evoked potentials

Eight cases had detectable Brainstem auditory evoked potentials, with 6 had prolonged or loss of later waves (5, 9, 10, 14, 21) and 2 had the normal response (13, 16). Visual evoked potentials (VEP) were performed in 3 cases and all reported prolonged VEP (5, 16). Somatosensory evoked potentials (SEP) was performed in 2 cases and all reported prolonged SEP (13, 14).

## Discussion

To the best of our knowledge, this is the first systematic investigation of electrophysiological features in CMTX1 with transient central nervous deficits. In the present study, we found that all cases had significant but variable degrees of slowed nerve conduction. The median MNCV was the most frequently detected and valuable parameter.

Onset of CMTX1 with transient central nervous system deficits occurs most often in early childhood (4). Typical features of CMTX1 disease may not be recognized until after presentation with central nervous system manifestations, such as recurrent and transient weakness, dysarthria, and dysphagia (4). CMTX1 is usually caused by mutation in the *GJB1* gene encoding the gap junction beta 1 protein connexin 32 (Cx 32) (3). Proposed mechanisms include loss of Cx32 function affecting the gap junctions in the myelin sheath and causing CMTX1 peripheral manifestations or gain of function affecting the central nervous system (2). The pathological mechanism by which *GJB1* mutations cause the CNS deficit in patients with CMTX1 is not well understood, but may involve a decrease in the number of functioning gap junctions between oligodendrocytes and astrocytes, disrupting gap junction communication and leading to abnormalities in the ability of these cells to regulate intercellular exchange of ions and small molecules (2). It is important to consider CMTX1 in previously healthy children who present with symptoms suggestive of recurrent or transient central nervous system deficits and to conduct thorough examination for features of CMT disease and electrophysiological testing (4).

Median MNCV can be a sensitive parameter to support the diagnosis of CMTX1 with transient central nervous system deficits. In the current study, NCVs at presentation were slower and characterized as intermediate (30–40 m/s) or mild (>40 m/s) slowing in CMTX1 patients with transient central nervous system deficits. Transient paralysis in children may also represent a feature of mitochondrial encephalopathy with lactic acidosis and stroke-like episodes (MELAS), ADEM, adrenoleukodystrophy, moya moya disease, and younger transient ischemic attack (TIA). Electrodiagnostic irregularities noted at presentation with central nervous system deficits are expected in a chronic hereditary motor and sensory neuropathy, as documented in the present analysis (4).

Uniform slowing of NCVs is suggestive of hereditary demyelinating neuropathy. So NCVs are valuable to separate demyelinating and axonal damage in CMTX1. Theoretically, CMTX1 is characterized by sensory-motor neuropathy with mixed axonal and demyelinating pathology. In the present study, electrophysiological findings during the episode of acute nervous system deficits showed a diffuse and symmetrical slowing of MNCV and SNCV consistent with demyelinating-type neuropathy (6). Reduced CMAP and SNAP amplitudes and prolonged distal latencies were also present. One month following the episode, the MNCV, SNCV, and distal latencies were unchanged, showing evidence of peripheral involvement during the chronic disease (6).

Prolonged brainstem auditory evoked potentials, VEP and SEP were found in most CMTX1 patients with transient central nervous deficits. It would be interesting to know the presence of subclinical alterations of the central white matter before the acute event.

## Conclusions

This case series serves as a reminder that the differential diagnosis of acute lesions of cerebral white matter should include the possibility of CMTX disease. Electrophysiological examination might be helpful in the diagnosis of recurring and intermittent neurological deficits with white matter lesion and median MCV is a sensitive and valuable parameter.

## Author contributions

QW design, acquisition of data, analysis and interpretation of data, drafting and revision of article, final approval. LC acquisition of data, analysis and interpretation of data, final approval. YX design, drafting and revision of article, final approval. CY design, acquisition of data, analysis and interpretation of data, drafting and revision of article, final approval.

### Conflict of interest statement

The authors declare that the research was conducted in the absence of any commercial or financial relationships that could be construed as a potential conflict of interest.

## References

[1] Berciano J García A Gallardo E Peeters K Pelayo-Negro AL Álvarez-Paradelo S. Intermediate charcot-marie-tooth disease: an electrophysiological reappraisal and systematic review. J Neurol. (2017) 264:1655–77. 10.1007/s00415-017-8474-3 28364294

[2] Wang Y Yin F. A review of X-linked charcot-marie-tooth disease. J Child Neurol. (2016) 31:761–72. 10.1177/0883073815604227 26385972

[3] Liu L Li XB Hu ZHM Zi XH Zhao X Xie YZ. Phenotypes and cellular effects of GJB1 mutations causing CMT1X in a cohort of 226 Chinese CMT families. Clin Genet. (2017) 91:881–91. 10.1111/cge.12913 27804109

[4] Al-Mateen M Craig AK Chance PF. The central nervous system phenotype of X-linked charcot-marie-tooth disease: a transient disorder of children and young adults. J Child Neurol. (2014) 29:342–8. 10.1177/0883073812474343 23400245

[5] Panas M Kalfakis N Karadimas C Vassilopoulos D. Episodes of generalized weakness in two sibs with the C164T mutation of the connexin 32 gene. Neurology (2001) 57:1906–8. 10.1212/WNL.57.10.1906 11723288

[6] Wu N Said S Sabat S Wicklund M Stahl MC. Recurrent episodes of stroke-like symptoms in a patient with charcot-marie-tooth neuropathy X type 1. Case Rep Neurol. (2015) 7:247–52. 10.1159/000442410 26955336 PMC4777946

[7] Kim GH Kim KM Suh SI Ki CS Eun BL. Charcot-marie-tooth disease masquerading as acutedemyelinating encephalomyelitis-like illness. Pediatrics (2014) 134:e270-3. 10.1542/peds.2012-3243 24958582

[8] Liberati A Altman DG Tetzlaff J Mulrow C Gotzsche PC Ioannidis JP. The PRISMA statement for reporting systematic reviews and meta-analyses of studies that evaluate health care interventions: explanation and elaboration. J Clin Epidemiol. (2009) 62:e1–34. 10.1136/bmj.b2700 19631507

[9] Hanemann CO Bergmann C Senderek J Zerres K Sperfeld AD. Transient, recurrent, white matter lesions in X-linked Charcot-Marie-Tooth disease with novel connexin 32 mutation. Arch Neurol. (2003) 60:605–9. 10.1001/archneur.60.4.605 12707076

[10] Fusco C Frattini D Pisani F Spaggiari F Ferlini A Della Giustina E. Coexistent central and peripheral nervous system involvement in a Charcot-Marie-Tooth syndrome X-linked patient. J Child Neurol. (2010) 25:759–63. 10.1177/0883073809344119 20382840

[11] Sakaguchi H Yamashita S Miura A Hirahara T Kimura E Maeda Y. A novel GJB1 frameshift mutation produces a transient CNSsymptom of X-linked Charcot-Marie-Tooth disease. J Neurol. (2011) 258:284–90. 10.1007/s00415-010-5752-8 20857133

[12] Zhong L Yan K Liu C Xue J Wu L Yin F. Clinical reasoning: a young man with reversible paralysis, cerebral white matter lesions, and peripheral neuropathy. Neurology (2012) 79:e70-2. 10.1212/WNL.0b013e3182661eca 22915180

[13] Sagnelli A Piscosquito G Chiapparini L Ciano C Salsano E Saveri P. X-linked Charcot-Marie-Tooth type 1: stroke-like presentation of a novel GJB1 mutation. J Peripher Nerv Syst. (2014) 19:183–6. 10.1111/jns5.12070 24863494

[14] Zhao Y Xie Y Zhu X Wang H Li Y Li J Transient, recurrent, white matter lesions in x-linked Charcot-Marie-tooth disease with novel mutation of gap junction proteinbeta 1 gene in China: a case report. BMC Neurol. (2014) 14:156 10.1186/s12883-014-0156-5 25086786 PMC4131157

[15] McKinney JL De Los Reyes EC Lo WD Flanigan KM. Recurrent central nervous system white matter changes in charcot-Marie-tooth type X disease. Muscle Nerve (2014) 49:451–4. 10.1002/mus.24108 24170412

[16] Kulkarni GB Mailankody P Isnwara PP Prasad C Mustare V. Episodic neurological dysfunction in hereditary peripheral neuropathy. Ann Indian Acad Neurol. (2015) 18:111–4. 10.4103/0972-2327.144314 25745327 PMC4350196

[17] Xie C Zhou X Zhu D Liu W Wang X Yang H. CNS involvement in CMTX1 caused by a novel connexin 32mutation: a 6-year follow-up in neuroimaging and nerveconduction. Neurol Sci. (2016) 37:1063–70. 10.1007/s10072-016-2537-6 27098243

[18] Schelhaas HJ Van Engelen BG Gabreëls-Festen AA Hageman G Vliegen JHR van der Knaap MS Transient cerebral white matter lesions in a patient with connexin32 missense mutation. Neurology (2002) 59:2007–8. 10.1212/01.WNL.0000038390.29853.46 12499506

[19] Halbrich M Barnes J Bunge M Joshi C. A V139M mutation also causes the reversible CNS phenotype in CMTX. Can J Neurol Sci. (2008) 35:372–4. 10.1017/S0317167100008994 18714809

[20] Rosser T Muir J Panigrahy A Baldwin EE Boles RG. Transient leukoencephalopathy associated with X-linkedCharcot-Marie-Tooth disease. J Child Neurol. (2010) 25:1013–6. 10.1177/0883073809352378 20472869

[21] U-King-Im JM Yiu E Donner EJ Shroff M MRI findings in X-linked Charcot-Marie-Tooth disease associatedwith a novel connexin 32 mutation. Clin Radiol. (2011) 66:471–4. 10.1016/j.crad.2010.11.009 21300330

