# Supplementary material for: The Electrophysiological Features in X-Linked Charcot-Marie-Tooth Disease With Transient Central Nervous System Deficits
Source: Front Neurol. 2018 Jun 27;9:461. doi: 10.3389/fneur.2018.00461 (PMC6036262; doi:10.3389/fneur.2018.00461)
Supplement: Supplementary file 1 [file Data_Sheet_1.docx]

**Supplement table** electrophysiological features in CMTX1 with transient central nervous system deficits

| **No. of patient** | **median nerve** | | | | | **ulnar nerve** | | | | | **peroneal nerve** | | | **tibial nerve** | | | **sural nerve** | |
| --- | --- | --- | --- | --- | --- | --- | --- | --- | --- | --- | --- | --- | --- | --- | --- | --- | --- | --- |
|  | **CMAP Amplitude (mv)** | **Distal Latency (ms)** | **MNCV (m/s)** | **SNAP Amplitude (uV)** | **SNCV (m/s)** | **CMAP Amplitude (mv)** | **Distal Latency (ms)** | **MNCV (m/s)** | **SNAP Amplitude (uV)** | **SNCV (m/s)** | **CMAP Amplitude (mv)** | **Distal Latency (ms)** | **MNCV (m/s)** | **CMAP Amplitude (mv)** | **Distal Latency (ms)** | **MNCV (m/s)** | **SNAP Amplitude (uV)** | **SNCV (m/s)** |
| **1** | 0.9 | 4.6 | 32 |  |  |  |  |  |  |  | 0.6 | 5.2 | 31 |  |  |  | 5 | 32 |
| **2** | 3.6 | 4.2 | 35 |  |  |  |  |  |  |  | 2.7 | 7.1 | 37 |  |  |  | 3 | 31 |
| **3** |  |  |  |  |  |  |  |  |  |  |  |  |  | 8.1 (> 5) |  | 33.2 (> 45.5) | 10 (> 6) |  |
| **4** |  |  |  |  |  |  |  |  |  |  |  |  |  | 5.2 (> 5) |  | 32.5 (> 45.5) | 6.5 (> 6) |  |
| **5** | 2(>5) | 6(<3) | 35(>45) | 10 (>15) | 36(>45) |  |  |  | 5(>10) | 34(>45) | 2.4(>3) | 4(<3.2) | 33(>40) | 4.7 (> 5) | 6 (<4) | 33 (> 40) |  |  |
| **6** | 0.59(>3) | 5.8(<4.6) | 36.9(>49.5) | 2 (>7) | 40(>47.1) | 2.1(>5) | 3.8(<3.8) | 37(>49.9) | 3.1(>6.9) | 41.1(>46.8) | 0.08(>4) | 6.9(<6.8) | 30.8(>42.7) | 0.55 (> 4.3) | 6.8 (<5.7) | 35.4 (> 41.6) | 3.4(>7.4) | 32.9(>40.7) |
| **7** | 0.4(>3) | 10.6(<3.2) | 40.4(>45) | 6.3 (>15) |  | 0.4(>3) | 8.7(<3.2) | 36.8(>40) | 6.2(>15) |  | 0.19(>5) | 19.2(<3) | 30.5(>45) | 0.72 (> 5) | 20.6 (<3) | 25.1 (> 45) |  |  |
| **8** |  |  | 40 |  | 32 |  |  | 37 |  |  |  |  | 36 |  |  | 37 |  | 32 |
| **9** | 1.4 |  | 37 | absent | decrease | 13 |  | decrease | 2.2 | decrease |  |  |  |  |  |  |  |  |
| **10** | 1.7 decrease 92% | 4.2 increase | 40 decrease | 3.2 decrease | 33 decrease | 8.9 normal | 2.7 normal | 33 decrease | 1.8 decrease | 36 decrease |  |  |  | 0.3 decrease | 4.5 normal | absent |  |  |
| **11** | 1.0(>3.5) | 2.2(<2.4) | 36.8(>48) | 20.5 (>20) | 42.9(>44) |  |  |  |  |  |  |  |  | 6.8 (> 6) | 1.9 (<2.9) | 28.1 (> 41) | 5.3(>10) | 29.9(>41) |
| **12** | low |  | 37 | low | 38 |  |  |  |  |  |  |  |  |  |  |  |  |  |
| **13** |  | mild prolonged | 34 | absent |  |  |  |  |  |  |  |  |  |  |  |  |  |  |
| **14** | 10.6 |  | 48.5 | 4.6 | 50.2 | 8 |  | 48 | 3 | 48.4 | 1.4 |  | 33.8 | 0.3 |  | 38.7 | 8.8 | 42 |
| **15** | reduced | prolonged | slow | reduced | slow | reduced | prolonged | slow | reduced | slow |  |  |  |  |  |  |  |  |
| **16** | a sensorimotor demyelinating and axonal neuropathy | | | | | | | | | | | | | | | | | |
| **17** | a sensorimotor demyelinating and axonal neuropathy | | | | | | | | | | | | | | | | | |
| **18** |  |  |  |  |  |  |  |  |  |  |  |  |  |  |  | slow |  | slow |
| **19** |  |  | slow |  |  |  |  |  |  |  |  |  |  |  |  |  |  |  |
| **20** | abnormal, demyelinating sensorimotor neuropathy, tibialis | | | | | | | | | | | | | | | | | |
| **21** | slow MCV and absent SNAP | | | | | | | | | | | | | | | | | |

CMTX: X-linked Charcot-Marie-Tooth disease, CMAP: compound muscle action potential, MNCV: median motor nerve conduction velocity, SNAP: sensory nerve action potential (SNAP), SNCV: sensory nerve conduction velocity
